# Examining Tensions That Affect the Evaluation of Technology in Health Care: Considerations for System Decision Makers From the Perspective of Industry and Evaluators

**DOI:** 10.2196/medinform.8207

**Published:** 2017-12-08

**Authors:** Laura Desveaux, James Shaw, Ross Wallace, Onil Bhattacharyya, R Sacha Bhatia, Trevor Jamieson

**Affiliations:** ^1^ Institute for Health Systems Solutions and Virtual Care Women's College Hospital Toronto, ON Canada; ^2^ Institute for Health Policy, Management, and Evaluation University of Toronto Toronto, ON Canada; ^3^ Santis Health Toronto, ON Canada; ^4^ Department of Family and Community Medicine University of Toronto Toronto, ON Canada; ^5^ Department of Medicine University of Toronto Toronto, ON Canada; ^6^ Division of General Internal Medicine St. Michael's Hospital Toronto, ON Canada

**Keywords:** technology, evaluation, policy, healthcare

## Abstract

Virtual technologies have the potential to mitigate a range of challenges for health care systems. Despite the widespread use of mobile devices in everyday life, they currently have a limited role in health service delivery and clinical care. Efforts to integrate the fast-paced consumer technology market with health care delivery exposes tensions among patients, providers, vendors, evaluators, and system decision makers. This paper explores the key tensions between the high bar for evidence prior to market approval that guides health care regulatory decisions and the “fail fast” reality of the technology industry. We examine three core tensions: balancing user needs versus system needs, rigor versus responsiveness, and the role of pre- versus postmarket evidence generation. We use these to elaborate on the structure and appropriateness of evaluation mechanisms for virtual care solutions. Virtual technologies provide a foundation for personalized, patient-centered medicine on the user side, coupled with a broader understanding of impact on the system side. However, mechanisms for stakeholder discussion are needed to clarify the nature of the health technology marketplace and the drivers of evaluation priorities.

## Introduction

Providing patient-centered care is an ongoing challenging due to rising costs [1], poor access [2], increasing complexity of patient needs [3], and the provider-centered structure of health systems [4]. Virtual care technologies have the potential to mitigate these challenges by lowering costs, improving access, and managing complexity [5-7], while being tailored to the needs and wants of users. These technologies can also support population-level research and the application of scientific evidence at a system level by providing real-time access to data across a broad population [8]. Despite this, the uptake of both provider- and patient-facing technologies has been limited in health systems compared to many other industries [9,10]. However, the mobile devices needed to access virtual care technologies are already in the hands of most individuals [11], which raises the question “What is limiting their potential in health care”?

The traditional approach of evidence-based medicine is at odds with the “fail fast” mentality of the technology industry, where rapid iterative testing facilitates early feedback from users leading to course corrections and better solutions [12]. Potential health care disrupters are confronted with a web of regulations, contractual obligations, provider interests, and interlocking financial incentives [10]. Some of the most challenging roadblocks are related to safety concerns and risk management; just because people like and use certain health-related apps does not mean they are safe and achieve positive health-related outcomes. To address questions of safety and effectiveness, pharmaceutical and medical device industries have established evaluation paradigms [13,14]. However, these approaches may not be appropriate for virtual care solutions because their high cost, long timelines, and rigid protocols do not account for the dynamic nature of software and the speed of the technology marketplace [15,16].

Efforts to integrate the high-paced consumer technology market with health care delivery exposes tensions at the intersection of users (including patients and health care providers), vendors, third-party evaluators (including scientific researchers), and system decision makers. The objective of this paper is to explore the key tensions between the high bar for evidence prior to market approval that guides health care regulatory decisions and the “fail fast” reality of the technology industry. We then elaborate on the implications of these tensions on the structure and appropriateness of evaluation mechanisms for virtual care solutions. Our goal is to carefully examine three core tensions: (1) balancing user needs versus system needs, (2) rigor versus responsiveness, and (3) the role of pre- versus postmarket evidence generation—the latter exploring the extent to which evidence of effectiveness and/or safety should be (and can be accurately) demonstrated before a product is used in real life. These observations come from our experiences with virtual care implementation and evaluation, including large randomized controlled trials (RCTs) [17-19], consultation with technology start-ups through the Canadian Government’s Industrial Research Assistance Program [20], and dialogue with policy stakeholders [21].

The integration of virtual care “is hampered because different stakeholders hold different assumptions, values and world views, ‘talk past’ each other, and compete for recognition and resources” [22]. Though stakeholder engagement has long been proposed in system design, it is not routinely done in health care. Mechanisms for more effective stakeholder dialogue are needed in order to establish a common vision, including consensus on what constitutes value, how it is determined, and who should be the primary beneficiaries. The presentation of the following tensions is intended to both illuminate these problems and facilitate this dialogue.

## Should Technologies and Evaluations Prioritize User Needs Versus System Needs?

Policy making for virtual care technologies must balance several priorities, such as economic and health care objectives [23]. Economic policy defines success as the creation of jobs, with the assumption that virtual care innovation “paves the road to economic development, solving societal challenges along the way” [23]. This favors economic interest over health system objectives, creating a tension between a system designed to facilitate innovation and a system that benefits its users. Health technology assessment (HTA) emerged as an approach to provide information about the efficacy, safety, and cost-effectiveness of health technologies for the purposes of decision making. Although its application aims to initiate processes that support the institutionalization of virtual care solutions, few examples of HTA demonstrate a commitment to understanding the needs, realities, and practices of end users [24]. HTA has standardized the value of different outcomes but does not address how a given solution fits with the end user’s reality. In short, HTA is primarily devoted to the needs of policy makers, as opposed to the needs of end users.

The consumer-oriented nature of virtual care allows for customization to the specific needs of the user, resulting in a wide range of vendors, user interface options, mobile apps, and wearables. These solutions can be rapidly developed and distributed, and they are easy to modify based on ongoing feedback from users, generating products that are tailored to user priorities, thus increasing the likelihood of adoption and meaningful use. However, focusing primarily on local user priorities can lead to solutions that are developed without considering system-level priorities, such as interoperability, change management, system-level cost-effectiveness, and population-level outcomes. As a consequence, many pilots of virtual care technologies have shown local effectiveness across a variety of clinical areas but fail to be used widely in practice [25,26]. In contrast, the development of universal solutions that meet industry standards and are widely implemented often occurs at the expense of responsiveness to diverse user needs across different contexts.

We suggest that a stronger consideration of system needs (ie, how virtual care technologies will function within the context of the larger system) while incorporating user needs, priorities, and values, will facilitate widespread adoption. This could be done by (1) identifying how a virtual solution fits within a larger system strategy, (2) developing a targeted outcome assessment that reflects both user and system needs, and (3) creating a change management plan that considers contextual factors to support rapid scale-up of successful interventions. It is worth noting that “scaling” in this context does not necessarily imply replication, but rather the facilitated diffusion (and evaluation) of solutions from one setting to another with the flexibility to allow for tailoring and modification.

Despite the range of perspectives, it is important to acknowledge that when it comes to the implementation of new technologies into organizations and systems, context and culture tend to drive changes in the form and use of technology, rather than the other way around [29]. This underscores the critical role of evaluation to help understand the local realities that may explain observed effects, unanticipated harms, or lead to broad rejection of the technology altogether. For example, a systematic review of 37 interventions revealed that suboptimal implementation was explained by the lack of attention to (1) specifying the purposes and benefits of virtual solutions and establishing their value to users, (2) effects on roles and responsibilities, (3) risk management, and (4) using user knowledge to modify implementation processes [30]. Technology may fail to become adopted when users do not perceive that the organization has a culture that is supportive of change, the solution does not align with perceived organizational priorities, and the impact of using the technology on individual accountability and liability is not understood [31].

## Do Evaluations Prioritize Rigor Versus Responsiveness?

Technology is dynamic and easy to modify on an ongoing basis. Approaching evaluation as if virtual care technologies are a static intervention may lead to the perception of more rigor, but the results may ultimately be rendered obsolete in light of updates to the technology itself. The rigor required for market entry in health care is a key element of regulatory systems, highlighting the need for system decision makers to consider the estimated opportunity costs, financial costs, and potential harms of a more open or closed market. Unlike pharmaceuticals, virtual care technologies often exist simultaneously in both the health care and consumer device marketplaces. These intertwined marketplaces must necessarily influence regulations and requirements that govern entry into the health care marketplace. The risk of rigorous evaluations with lengthy timelines is that health care technologies become “fixed” relative to the consumer marketplace, resulting in a confusing mismatch for the end user between technologies that do similar things. The risk of a marketplace without constraints to entry is the proliferation of technologies of uncertain value. In the best case, they may be inexpensive and outcome neutral; in the worst case, they may be costly and have an adverse effect on health care quality.

Many virtual care solutions can collect and transmit data, so it is possible to continuously monitor safety and respond quickly. In a study among patients with heart failure, remote monitoring ensured the timely transmission of data to the health care team, resulting in early intervention as needed and a 3% mortality rate in the intervention group (compared to 8.2% among controls) [32]. Extracting user data from devices automatically as a condition of market entry can alleviate issues of access and data reconciliation that plague health outcomes research [33,34]. This supports evaluations that move beyond the traditional RCT model towards a more adaptive model [35,36], better positioning them to balance both rigor and responsiveness. Unlike traditional RCTs, adaptive trial design allows for modifications to the trial after its initiation, including aspects such as target population, intervention design, dose, duration, and statistical procedures [37]. Adaptive trials maintain validity and integrity by ensuring modifications occur in response to observations made during the trial and prior to the unblinded analysis of trial data.

Pre-market evaluations employing rapid, iterative cycles are well suited to support this model [38]. These types of solutions would parallel existing models in quality improvement, allowing for deployment, monitoring, incremental improvement, and local tailoring. Although this does not eliminate the need to define requirements for evaluation rigor, it provides some reassurance that pre-market rigor can be compromised without the threat of long-term negative consequences. It is also important to note that while harnessing the collection of simple, patient-generated data expedites the timeliness of ongoing evaluation and improvement cycles, it requires clarity around the nature of “ownership” and the appropriate mechanisms to ensure security of the data [39].

## Is the Evaluation Structure Influenced by Pre- or Postmarket Status?

Pharmaceuticals and medical devices are subject to pre-market evaluations to establish their suitability for entry into the broader health system. Although this assumption has carried over into the domain of virtual care, the extent to which pre-market evaluation can truly establish safety and effectiveness for these dynamic solutions remains unclear. Virtual solutions have the added advantage of flexibility when compared to pharmaceuticals, as regular updates enable modifications to address safety concerns (as opposed to being taken off the market entirely).

Safety in virtual care extends far beyond the typical health care considerations of morbidity and mortality. Direct users of virtual care technologies may experience loss of privacy, poor data quality, and suboptimal clinical-decision support. The latter extends from mild and relatively inconsequential decisions (eg, being advised of a suboptimal exercise scheme) to harmful and irreversible clinical actions (eg, being advised to take a harmful dose of insulin) [40]. A recent Institute of Medicine report outlined such safety concerns but did not uncover evidence of significant issues [41]. The majority of evaluators held the opinion that strict regulation would greatly stifle innovation in this space [41].

Current evaluation standards stem from pharmaceutical and medical device industries, where health evidence is generated through rigorous, (ideally) randomized and controlled evaluations that demonstrate both safety and effectiveness prior to use in a real-world setting [13,14]. Such controlled studies are generalizable and can apply across a diverse array of settings because the intervention (eg, medication or a medical device) does not interact substantially with an array of external, contextual factors. However, due to the complex adaptive nature of sociotechnical systems (in which virtual care technologies must fit) [42], safety and effectiveness cannot be determined in a pre-market vacuum. They depend on a range of factors, including interaction between the technology, users, organizations, and environmental conditions that vary across sites [43]. Simply put, the “intervention” in any local environment is the intersection of all of these components. “Idealized” and controlled evaluations therefore *limit* generalizability with respect to virtual care technologies, arguably wasting valuable time and resources, whereas real-life interplay truly determines whether a solution works.

The overarching theme throughout this dialogue is *complexity* in a system of intersecting priorities, within which virtual care technologies can act as a disruptive catalyst. System tolerance for risk in health care has historically been quite low, resulting in strict requirements for market entry; however, the presence of technology is slowly increasing individual risk tolerance for readily available solutions. This reality may signal an inevitable shift in focus from dimensions of safety and effectiveness to the balanced evaluation of “value-add” for end users. If establishing effectiveness is the priority, the outcome of interest will dictate requirements for recruitment and adequate exposure time, in order to reliably detect a meaningful difference. Ultimately, the nature of the market and the degree of regulation will inform the extent to which safety, effectiveness, or user preference drives the uptake of virtual care solutions and the role that evaluation plays within it.

## Conclusion

A recurring theme across our scientific and industry-based engagements has been the challenge faced by technology vendors when attempting to define their customer (defined here as the individual who will purchase their technology). Owing to the relatively open nature of the consumer marketplace, many vendors simultaneously market their virtual care solution to patients, clinicians, clinics, and the government. This reality highlights the failure of the system to clearly and explicitly define the health care marketplace, including the regulations and evaluation parameters required for entry. This is compounded by the fact that, in both single- and multipayer health care systems, the health care “payer” is rarely the end user.

It is important to note that we have simplified these considerations by presenting them as distinct tensions, whereas the interplay across these categories in practice makes them difficult to disentangle. Systematic consideration of the questions arising from these tensions will help define the type of evaluation that meets the needs of both the system and its end users (see Table 1). At a higher level, it can support transformation by challenging the current structure of health care delivery that limits change [44]. Productive dialogue relies on collaborative relationships and requires that those involved acknowledge the range of priorities and accountabilities that operate across the system; but more importantly, they are essential in order to curtail fragmentation and define the appropriate yet comprehensive parameters of both the market and the role of evaluation within it. Stakeholder discussions are needed to clarify the nature of the health technology marketplace, for whom the marketplace primarily aims to generate value, and hence, the drivers of evaluation priorities. These discussions will inform how virtual care solutions are developed, evaluated, and incorporated into health care delivery.

Simply put, regulations that heavily prioritize the system risk rejection by end users, the development of workarounds, or suboptimal outcomes resulting from a failure to consider local context. In contrast, regulations that prioritize end users risk a degree of technological customization that exacerbates fragmentation within the system and is unlikely to improve overall health outcomes or costs. Similarly, strict requirements for pre-market assessment are likely to lead to overly general evaluation results that provide false reassurance, while sparse regulation may lead to the introduction of unsafe and ineffective tools. However, the technologies themselves provide a promising return in exchange for navigating these complex tensions. The plethora of data they generate provides a foundation for personalized, patient-centered medicine on the user side, coupled with a broader understanding of impact on the system side.

**Table 1:** Tensions and underlying key questions.

Tension	Questions
Prioritizing user needs versus system needs	How does a local strategy fit within the larger system?
	What are the relevant outcomes that reflect user and system needs?
	How can system infrastructure support the scale of successful solutions?
Prioritizing rigor versus responsiveness	How does the consumer virtual care marketplace influence the health care marketplace?
	What outcomes require a rigorous approach?
	What infrastructure is needed to support real-time consolidation and analysis of data?
Pre- or postmarket status influence on evaluation structure	What is the minimum requirement for system entry?
	What are the appropriate pathways for solutions to enter the health care system?
	How can we embed ongoing monitoring and evaluation alongside the use of virtual care solutions?

## Abbreviations

HTA: health technology assessment
RCT: randomized controlled trial
